# Prostaglandin E_2_ and its receptor EP2 trigger signaling that contributes to YAP‐mediated cell competition

**DOI:** 10.1111/gtc.12750

**Published:** 2020-02-07

**Authors:** Erika Ishihara, Yuya Nagaoka, Toshiaki Okuno, Satoshi Kofuji, Mari Ishigami‐Yuasa, Hiroyuki Kagechika, Kenya Kamimura, Shuji Terai, Takehiko Yokomizo, Yukihiko Sugimoto, Yasuyuki Fujita, Akira Suzuki, Hiroshi Nishina

**Affiliations:** ^1^ Department of Developmental and Regenerative Biology Medical Research Institute Tokyo Medical and Dental University (TMDU) Tokyo Japan; ^2^ Department of Biochemistry Juntendo University Graduate School of Medicine Tokyo Japan; ^3^ Institute of Biomaterials and Bioengineering Tokyo Medical and Dental University (TMDU) Tokyo Japan; ^4^ Division of Gastroenterology and Hepatology Graduate School of Medical and Dental Sciences Niigata University Niigata Japan; ^5^ Department of Pharmaceutical Biochemistry Graduate School of Pharmaceutical Sciences Kumamoto University Kumamoto Japan; ^6^ Division of Molecular Oncology Institute for Genetic Medicine Graduate School of Chemical Sciences and Engineering Hokkaido University Sapporo Japan; ^7^ Division of Molecular and Cellular Biology Kobe University Graduate School of Medicine Kobe Japan

**Keywords:** cell competition, COX‐2, E‐cadherin internalization, PGE_2_, YAP

## Abstract

Cell competition is a biological process by which unfit cells are eliminated from “cell society.” We previously showed that cultured mammalian epithelial Madin‐Darby canine kidney (MDCK) cells expressing constitutively active YAP were eliminated by apical extrusion when surrounded by “normal” MDCK cells. However, the molecular mechanism underlying the elimination of active YAP‐expressing cells was unknown. Here, we used high‐throughput chemical compound screening to identify cyclooxygenase‐2 (COX‐2) as a key molecule triggering cell competition. Our work shows that COX‐2‐mediated PGE_2_ secretion engages its receptor EP2 on abnormal and nearby normal cells. This engagement of EP2 triggers downstream signaling via an adenylyl cyclase‐cyclic AMP‐PKA pathway that, in the presence of active YAP, induces E‐cadherin internalization leading to apical extrusion. Thus, COX‐2‐induced PGE_2_ appears a warning signal to both abnormal and surrounding normal cells to drive cell competition.

## Graphical abstract

Mammalian epithelial Madin‐Darby canine kidney (MDCK) cells expressing constitutively active YAP are eliminated by apical extrusion when surrounded by “normal” MDCK cells. However, the molecular mechanism underlying it was unknown. We show that COX‐2‐induced PGE2 activates adenylyl cyclase‐cyclic AMP‐PKA signaling pathway that, in the presence of active YAP, induces E‐cadherin internalization leading to apical extrusion.

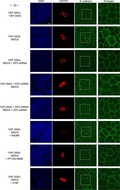

## 1 INTRODUCTION

“Cell competition” was first discovered in studies of cell–cell interactions in *Drosophila* (Morata & Ripoll, 1975). *Drosophila* cells heterozygous for the *Minute* mutation, which have reduced ribosomal activity, underwent apoptosis when confronted with wild‐type (WT) *Drosophila* cells. This observation led to the concept of “cell competition” in which a given cell compares its fitness to that of its neighboring cells. Cells with a relatively higher fitness level survive, whereas cells with a relatively lower fitness level are eliminated by either apoptosis or apical extrusion (Baker, 2017; de Beco, Ziosi, & Johnston, 2012; Bowling, Lawlor, & Rodriguez, 2019; Claveria & Torres, 2016; Madan, Gogna, & Moreno, 2018; Morata & Calleja, 2019; Wagstaff, Kolahgar, & Piddini, 2013). Cell competition is now a well‐established process among mammalian “cell societies” as well.

In *Drosophila*, cell competition can be induced by activation of proto‐oncogenes such as Myc, Ras and Src, as well as that of genes regulating cellular apicobasal polarity such as *Scribble* and *Discs large* (*Dlg*) (Baker, 2017; de Beco et al., 2012; Bowling et al., 2019; Claveria & Torres, 2016; Madan et al., 2018; Morata & Calleja, 2019; Nagata & Igaki, 2018; Wagstaff et al., 2013). In the developing mouse embryo, cells with low levels of Myc undergo apoptosis if in proximity to cells with higher Myc levels (Claveria, Giovinazzo, Sierra, & Torres, 2013; Sancho et al., 2013). Embryonic mouse cells carrying a heterozygous mutation of the riboprotein gene *Rpl24* also lose in competitions with embryonic cells bearing two WT *Rpl24* alleles (Oliver, Saunders, Tarle, & Glaser, 2004). In adult mouse tissues, cell competition has been induced by differences in Myc in cardiomyocytes, p53 in hematopoietic stem cells, Ras in intestinal epithelial cells and COL17A1 in mouse epidermal stem cells (Bondar & Medzhitov, 2010; Kon, 2018; Kon et al., 2017; Liu et al., 2019; Villa Del Campo, Claveria, Sierra, & Torres, 2014). Cell competition has also been observed in cultured Madin–Darby canine kidney (MDCK) epithelial cells. When MDCK cells expressing either of the oncogenic proteins Ras (G12V) or v‐Src are surrounded by nontransformed cells, the transformed MDCK cells are removed by apical extrusion (Hogan et al., 2009; Kajita et al., 2010; Maruyama & Fujita, 2017). Filamin and vimentin accumulate in the surrounding normal cells, whereas E‐cadherin is internalized in the apically extruded cells (Kajita et al., 2014; Saitoh et al., 2017). Thus, at least some mechanisms of cell competition induction are conserved from *Drosophila* to mammals.

Genetic screening studies in *Drosophila* have showed that activation of the transcriptional coactivator Yorkie (Yki) induces cell competition (Neto‐Silva, Beco, & Johnston, 2010; Tyler, Li, Zhuo, Pellock, & Baker, 2007; Ziosi et al., 2010). The mammalian homologue of Yki is Yes‐associated protein (YAP), which binds to TEA domain (TEAD) family transcription factors to initiate target gene expression (Meng, Moroishi, & Guan, 2016; Piccolo, Dupont, & Cordenonsi, 2014; Zheng & Pan, 2019). YAP activation is regulated by phosphorylation driven by signaling via the Hippo pathway. In response to Hippo signaling, five Ser residues of YAP are phosphorylated and YAP activity is suppressed. The YAP (5SA) mutant protein, in which these five key Ser residues are replaced with Ala, becomes constitutively active. In mouse fibroblast NIH3T3 cells, cell competition resulting in apoptosis was reportedly dependent on TEAD activity (Mamada, Sato, Ota, & Sasaki, 2015). We subsequently showed that MDCK cells and mouse hepatocytes also undergo YAP‐induced competition (Chiba et al., 2016; Miyamura et al., 2017). We generated doxycycline (Dox)‐inducible YAP (5SA)‐expressing MDCK cells [YAP (5SA) cells] and showed that they succumb to apical extrusion when surrounded by normal MDCK cells. This apical extrusion of YAP (5SA) cells was found to involve TEAD‐dependent gene expression, activation of the PI3K‐mTOR‐S6K pathway, actin polymerization and suppression of cell adhesion molecules such as fibronectin‐1 (Chiba et al., 2016; Nishio et al., 2019). However, the mechanism by which surrounding normal MDCK cells are able to recognize YAP (5SA) cells as abnormal and in need of removal by cell competition is unknown.

In this study, we established a high‐throughput chemical compound screening method to identify molecules contributing to the apical extrusion of YAP (5SA) cells. We show that COX‐2‐induced PGE_2_ serves as a warning signal to both abnormal and surrounding normal MDCK cells to drive cell competition.

## 2 RESULTS

### 2.1 A high‐throughput screening system can identify molecules involved in the apical extrusion of YAP (5SA) cells

**Figure 1:**
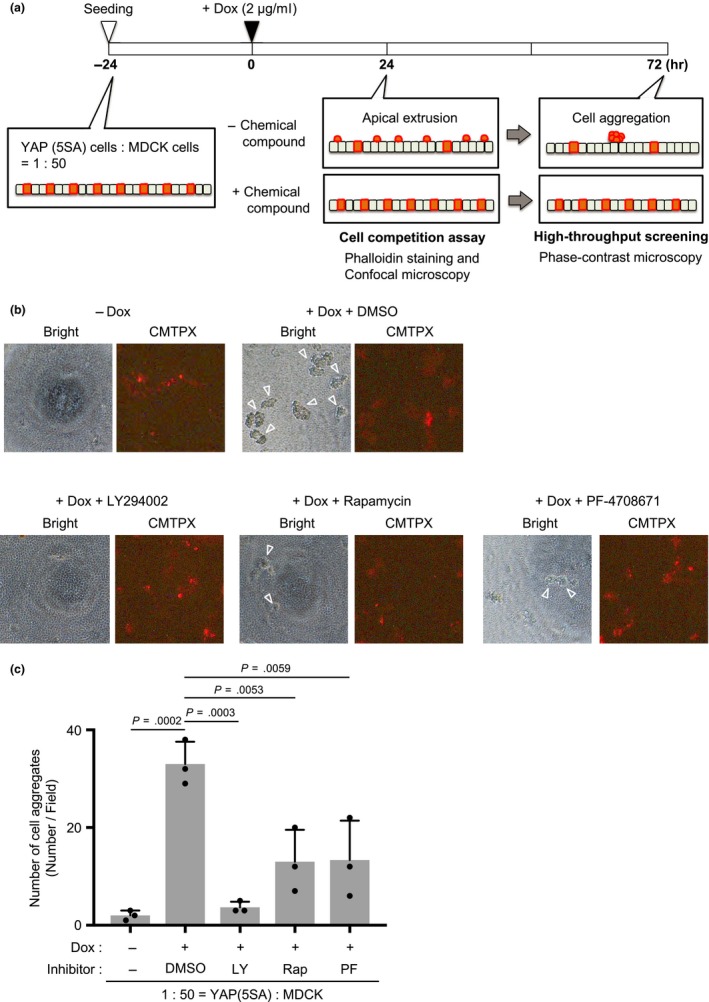
Standard cell competition assay and high‐throughput chemical compound screening. (a) Schematic diagram showing both the standard cell competition assay and the protocol used to screen for chemical compounds promoting the apical extrusion of YAP (5SA) cells during cell competition. In both cases, Dox‐inducible YAP (5SA) cells were labeled with CMTPX (red fluorescent dye), mixed 1:50 with unlabeled normal MDCK cells and incubated in cocultures for 24 hr. For standard cell competition assays, cocultures were incubated for another 24 hr with Dox before fixation. Labeled, apically extruded cells were detected by phalloidin staining and confocal microscopy. For high‐throughput chemical screening, cocultures were incubated for 72 hr with Dox with/without compounds before fixation. Aggregated cells were examined by phase‐contrast microscopy. (b) Representative images of cell aggregation in the high‐throughput screening protocol where CMTPX‐labeled YAP (5SA) cells were mixed 1:50 with unlabeled normal MDCK cells and treated (as indicated) for 72 hr with Dox plus LY294002 (LY, PI3K inhibitor), Rapamycin (Rap, mTOR inhibitor) or PF‐4708671 (PF, S6K inhibitor), starting at 24 hr postseeding. Controls were cocultures incubated for 72 hr without Dox or with Dox plus DMSO. “Bright,” phase‐contrast image. “CMTPX,” red fluorescence image. (c) Quantification of numbers of cell aggregates in the cocultures in (b). Data are the mean +*SD* (*n* = 3/group) of three independent experiments

To identify molecules involved in the apical extrusion of YAP (5SA) cells during cell competition, we sought to devise a method of high‐throughput screening. In our standard cell competition assay, YAP (5SA) cells are cocultured with normal MDCK cells at a ratio of 1:50 (Figure 1a). This cell mixture is treated with Dox at 24 hr postplating, and approximately 40% of YAP (5SA) cells in the coculture undergo apical extrusion at 24 hr post‐Dox. Apical extrusion is then confirmed by phalloidin staining of actin and confocal microscopy. However, these procedures are relatively complex and time‐consuming, and so not suitable for high‐throughput screening. We observed that, if our standard competition cultures were allowed to grow until 72 hr post‐Dox, many extruded YAP (5SA) cells floated up into the culture medium, but a significant percentage of these extruded cells remained attached and formed cell aggregates. These cell aggregates were easily observed using phase‐contrast fluorescence microscopy without staining (Figure 1a). This observation led us to suggest that cell aggregation might be useful as an index for high‐throughput screening of compounds inhibiting apical extrusion.

To investigate whether the cell aggregation we observed was indeed correlated with apical extrusion, we examined the effects of various apical extrusion inhibitors, including LY294002, an inhibitor of phosphoinositide‐3‐kinase (PI3K); rapamycin, an inhibitor of mammalian target of rapamycin (mTOR); and PF‐4708671, an inhibitor of p70S6 kinase (p70S6K), on cell aggregation after cell competition. We found that both apical extrusion and the formation of cell aggregates were suppressed by these three inhibitors (Figure 1b,c), showing that cell aggregation was indeed correlated with apical extrusion and validating the use of cell aggregate formation as a proxy for apical extrusion due to cell competition.

### 2.2 Cyclooxygenase‐2 is essential for the apical extrusion of YAP (5SA) MDCK cells

**Figure 2:**
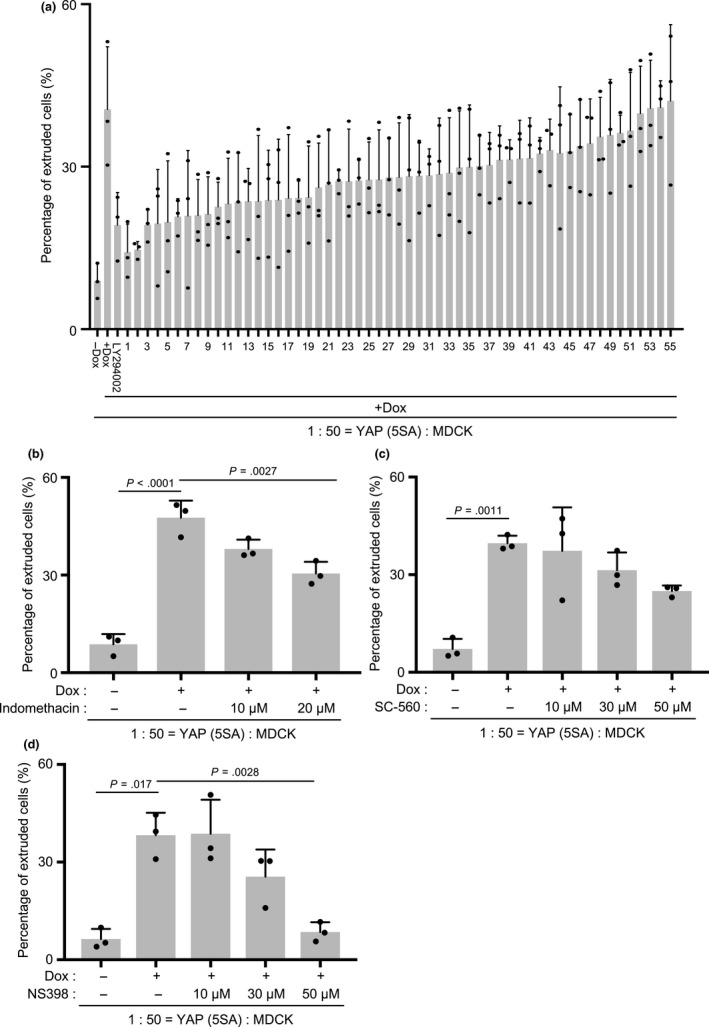
The effects of cyclooxygenase inhibitors on the apical extrusion of YAP (5SA) cells. (a‐d) Quantification of percentages of apically extruded cells in cocultures of labeled YAP (5SA) cells that were mixed 1:50 with unlabeled normal MDCK cells and incubated for 24 hr with Dox plus: (a) each of 55 chemical compounds or LY294002 (PI3K inhibitor), or the indicated concentrations of (b) indomethacin (COX‐1&2 inhibitor), (c) SC‐560 (COX‐1 inhibitor) or (d) NS398 (COX‐2 inhibitor). Controls were cocultures incubated with/without Dox in the absence of inhibitors. Data are the mean +* SD* (*n* = 3/group) of three independent experiments

We theorized that neighboring normal cells must receive some kind of chemical signal from YAP (5SA) cells that triggers the normal cells to initiate cell competition. To identify such molecules, we screened a library of 1,600 well‐characterized chemical compounds by examining cocultures of normal MDCK cells and YAP (5SA) cells at 72 hr post‐Dox (1st screening). We identified 75 chemical compounds that inhibited the formation of cell aggregates. Next, we used our standard cell competition assay to evaluate the effects of these 75 chemical compounds on YAP (5SA)‐induced apical extrusion at 24 hr post‐Dox (2nd screening). We observed that 55 chemical compounds induced cell aggregation without cell death (Figure 2a, Table S1). The top two inhibitory compounds were “harmine,” a drug for Parkinson's disease, and “chrysin,” an antifungal drug. A search of the literature showed that harmine induces the apoptosis of gastric cancer cells by down‐regulating cyclooxygenase‐2 (COX‐2) expression (Yu et al., 2016) and that chrysin inhibits COX‐2 expression in macrophages and induces Akt inactivation in U937 cells (Woo, Jeong, Inoue, Park, & Kwon, 2005). Thus, COX‐2 is a common target of harmine and chrysin. To investigate whether COX‐2 played a role in the apical extrusion of YAP (5SA) cells, we used our standard competition assay to examine the effects of indomethacin, an inhibitor of both COX‐1 and COX‐2; SC‐560, a selective COX‐1 inhibitor; and NS398, a selective COX‐2 inhibitor. Although indomethacin and SC‐560 partially inhibited the apical extrusion of cocultured YAP (5SA) cells (Figure 2b,c), 50 µM NS398 completely inhibited this process (Figure 2d). These results indicate that COX‐2 is essential for the apical extrusion of the YAP (5SA) cells cultured in competition with normal MDCK cells.

### 2.3 COX‐2 is not essential for the apical extrusion of K‐Ras (G12V) or v‐Src cells

**Figure 3:**
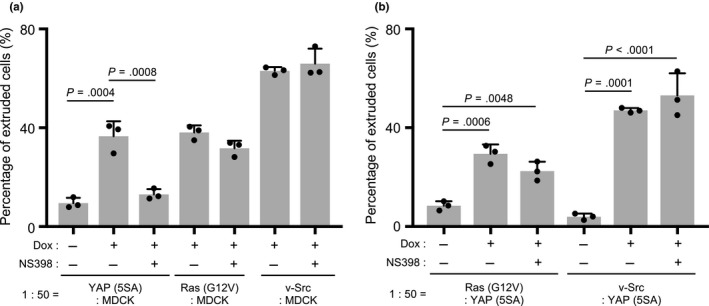
Effects of a COX‐2 inhibitor on the apical extrusion of Ras (G12V) or v‐Src cells. (a,b) Quantification of percentages of apically extruded cells in (a) cocultures of labeled YAP (5SA), Ras (G12V) or v‐Src cells that were mixed 1:50 with unlabeled normal MDCK cells, or (b) cocultures of labeled Ras (G12V) or v‐Src cells that were mixed 1:50 with unlabeled YAP (5SA) cells, and were treated with Dox plus NS398 (50 μM) for 24 hr starting at 24 hr postseeding. Controls were cocultures incubated for 24 hr with/without Dox in the absence of NS398. Data are the mean +* SD* (*n* = 3/group) of three independent experiments

As noted above, transformed MDCK cells expressing either K‐Ras (G12V) [Ras (G12V) cells] or v‐Src [v‐Src cells] under the control of Dox also lose in cell competitions with normal MDCK cells. To investigate whether COX‐2 was also involved in the apical extrusion of Ras (G12V) or v‐Src cells, we evaluated the effects of NS398 on cocultures of these cells with normal MDCK cells and compared them with YAP (5SA) cell cocultures. Strikingly, NS398 did not inhibit the apical extrusion of Ras (G12V) or v‐Src cells (Figure 3a). We previously reported that Ras (G12V) and v‐Src cells underwent apical extrusion when cocultured with YAP (5SA) cells (Chiba et al., 2016). To investigate whether COX‐2 allows YAP (5SA) cells to induce the apical extrusion of competitors, we examined the effects of NS398 on apical extrusion in cocultures of Ras (G12V) or v‐Src cells with YAP (5SA) cells. Once again, NS398 had no effect on the apical extrusion of the transformed MDCK cells (Figure 3b). Thus, COX‐2 participates in a mechanism that is specific to the apical extrusion of YAP (5SA) cells surrounded by normal MDCK cells.

### 2.4 PGE_2_ secretes into the culture medium during YAP (5SA) cell competition

**Figure 4:**
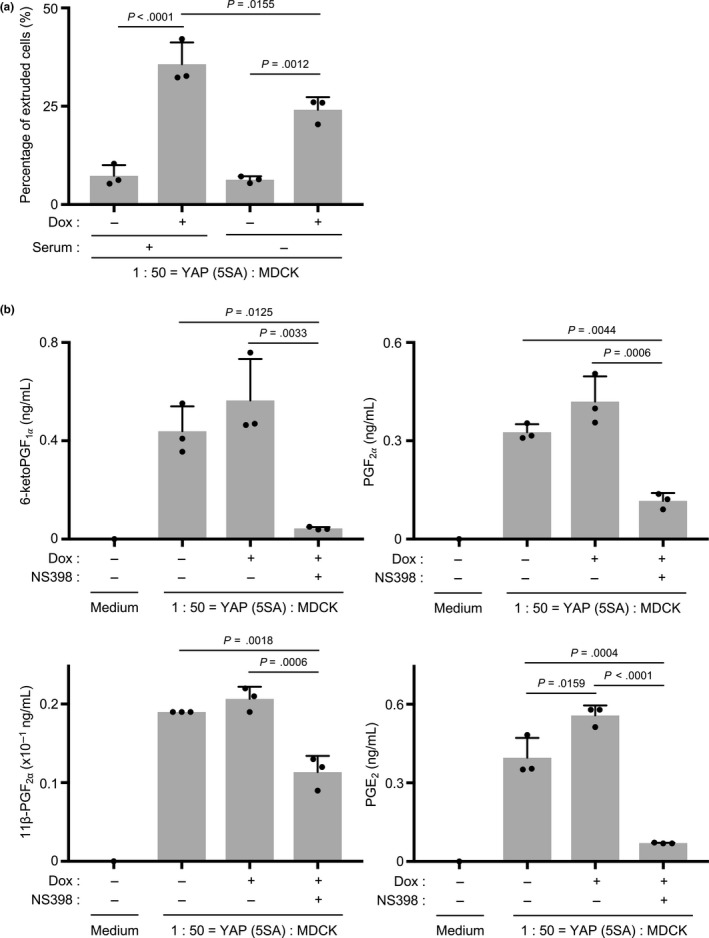
Secretion of COX‐2 products into the culture medium during YAP (5SA) cell competition. (a) Quantification of percentages of apically extruded cells in cocultures of labeled YAP (5SA) cells that were mixed 1:50 with unlabeled normal MDCK cells and incubated for 24 hr with/without Dox in serum‐free medium. Controls were cocultures incubated for 24 hr with/without Dox in standard culture medium containing FBS. Data are the mean +* SD* (*n* = 3/group) of three independent experiments. (b) Quantification of the indicated cyclooxygenase products, namely 6‐ketoPGF_1α_, PGF_2α_, 11β‐PGF_2α_ and PGE_2,_ released into the culture medium of cocultures of YAP (5SA) cells that were mixed 1:50 with normal MDCK cells and treated with/without Dox and with/without NS398 (50 μM) in serum‐free medium at 24 hr postseeding. The conditioned medium was collected at 24 hr post‐Dox addition. Control was medium alone lacking both Dox and NS398 (*n* = 1/group). Data of the experimental samples are the mean +* SD* (*n* = 3/group)

In our standard cell competition assay, conducted in culture medium containing fetal bovine serum (FBS), the percentage of YAP (5SA) cells that undergoes apical extrusion in the presence of normal MDCK cells but in the absence of Dox is typically 10%. Upon the addition of Dox (YAP activation), a marked increase to 35% of the YAP (5SA) cells in the coculture undergoes apical extrusion. However, because the presence of FBS in standard culture medium prevents the measurement of COX‐2 products by quantitative LC‐MS/MS analysis, we set up our competition cocultures in serum‐free medium lacking FBS. Upon the addition of Dox under these serum‐free conditions, the percentage of YAP (5SA) cells in the coculture that underwent apical extrusion was 25% (Figure 4a). We deemed this increase over the control level of 10% to be sufficient to determine differences in COX‐2 products upon YAP activation.

Next, we used quantitative LC‐MS/MS to analyze COX‐2 products in Dox‐treated, serum‐free cocultures of normal MDCK and YAP (5SA) cells. We detected significant levels of 6‐ketoPGF_1α_, which is a metabolite of prostaglandin (PG) I_2_; PGF_2α_; 11β‐PGF_2α_, which is a metabolite of PGD_2_; and PGE_2_ in the culture medium (Figure 4b). Among them, only PGE_2_ was induced by Dox treatment. Notably, the secretion of all four of these metabolites was suppressed by NS398. These results indicate that cell competition involving YAP (5SA) cells induces the secretion of the COX‐2 product PGE_2_.

### 2.5 PGE_2_ promotes the apical extrusion of YAP (5SA) cells

**Figure 5:**
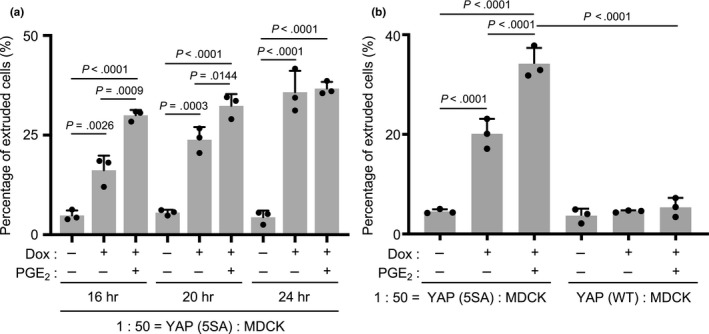
Effects of prostaglandin E_2_ on the apical extrusion of YAP (5SA) cells. (a) Quantification of percentages of apically extruded cells in cocultures of labeled YAP (5SA) cells that were mixed 1:50 with unlabeled normal MDCK cells and treated with Dox plus PGE_2_ (5 μM) for the indicated times starting at 24 hr postseeding. Controls were cocultures incubated with/without Dox in the absence of PGE_2_. (b) Quantification of percentages of apically extruded cells in cocultures of labeled YAP (5SA) or YAP (WT) cells that were mixed 1:50 with unlabeled normal MDCK cells. Cells were treated for 16 hr with Dox plus PGE_2_ (5 μM) starting at 24 hr postseeding. Controls were cocultures incubated for 16 hr with/without Dox in the absence of PGE_2_. For (a) and (b), data are the mean +* SD* (*n* = 3/group) of three independent experiments

To investigate whether PGE_2_ promotes the apical extrusion of YAP (5SA) cells, we conducted time‐course experiments. We examined the effects of PGE_2_ on the apical extrusion of YAP (5SA) cells at 16, 20 and 24 hr after the addition of Dox plus PGE_2_ to cocultures of normal MDCK and YAP (5SA) cells. Compared to control cocultures lacking PGE_2,_ addition of this metabolite significantly accelerated YAP (5SA) cell apical extrusion when measured at 16 or 20 hr post‐Dox, but that this difference had disappeared by 24 hr post‐Dox (Figure 5a). Next, we assessed whether PGE_2_ addition could induce the extrusion of MDCK cells expressing wild‐type YAP [YAP (WT) cells] when cocultured with normal MDCK cells. Although high expression of WT YAP protein is induced in these cells upon Dox treatment, this YAP is not activated. In fact, PGE_2_ could not induce the apical extrusion of YAP (WT) cells during cell competition (Figure 5b), indicating that PGE_2_'s role in inducing apical extrusion depends on YAP activation.

### 2.6 PGE_2_‐EP2 signaling induces the apical extrusion of YAP (5SA) cells

**Figure 6:**
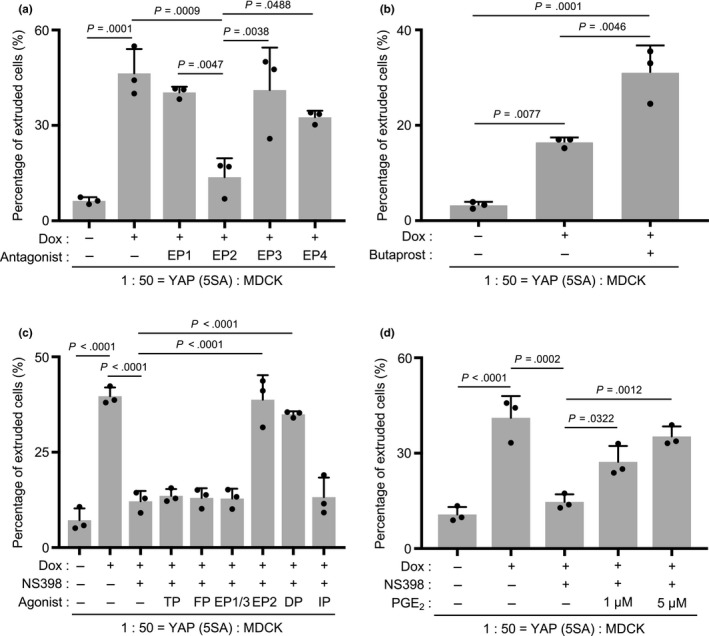
Effects of prostaglandin E_2_ and various receptor agonists or antagonists on the apical extrusion of YAP (5SA) cells. (a–d) Quantification of percentages of apically extruded cells in cocultures of labeled YAP (5SA) cells that were mixed 1:50 with unlabeled normal MDCK cells and treated with Dox plus the indicated compounds for various times starting at 24 hr postseeding. Controls were cocultures incubated for the appropriate times with/without Dox in the absence of the indicated compounds. (a) Cells were treated for 24 hr with Dox plus ONO‐8711 (EP1 antagonist, 10 μM), PF‐04418948 (EP2 antagonist, 10 μM), L‐798106 (EP3 antagonist, 10 μM) or ONO‐AE3‐208 (EP4 antagonist, 10 μM). (b) Cells were treated for 16 hr with Dox plus butaprost (EP2 agonist, 10 μM). (c) Cells were treated for 24 hr with Dox and NS398 (50 μM) plus butaprost (10 μM), U‐46619 (TP agonist, 100 nM), Fluprostenol (FP agonist, 10 μM), sulprostone (EP1/3 agonist, 10 μM), BW‐245C (DP agonist, 10 μM) or Cicaprost (IP agonist, 10 μM). (d) Cells were treated for 24 hr with Dox and NS398 (50 μM) plus PGE_2_ (1 µM or 5 µM). For (a)–(d), data are the mean +* SD* (*n* = 3/group) of three independent experiments

There are four PGE_2_ receptors, namely EP1, EP2, EP3 and EP4 (Park, Pillinger, & Abramson, 2006; Sugimoto & Narumiya, 2007). To determine which PGE_2_ receptor(s) was essential for the apical extrusion of YAP (5SA) cells, we examined the effects on this process of several selective antagonists: ONO‐8711 (EP1 antagonist), PF‐04418948 (EP2 antagonist), L‐798106 (EP3 antagonist) and ONO‐AE3‐208 (EP4 antagonist). The apical extrusion of YAP (5SA) cells cocultured with normal MDCK cells was completely inhibited by the EP2 antagonist, partially inhibited by the EP4 antagonist, but not inhibited by the EP1 and EP3 antagonists (Figure 6a). Conversely, to determine whether EP2 engagement and activation could promote the apical extrusion of YAP (5SA) cells, we applied the EP2 agonist butaprost to YAP (5SA): MDCK cocultures at 16 hr post‐Dox. Indeed, the percentage of YAP (5SA) cells that underwent apical extrusion was dramatically increased by this EP2 agonist (Figure 6b).

Next, to evaluate whether an EP2 agonist could reverse the impaired apical extrusion caused by COX‐2 inhibition, we treated YAP (5SA): MDCK cocultures with either butaprost (EP2 agonist), U‐46619 (TP agonist), Fluprostenol (FP agonist), sulprostone (EP1 and EP3 agonist), BW‐245C (DP agonist) or Cicaprost (IP agonist) in the presence of the COX‐2 inhibitor NS398. We observed that the suppression of YAP (5SA) cell apical extrusion induced by NS398 was rescued by the addition of either butaprost or BW‐245C (which triggers the same downstream signaling pathway), but not by the addition of U‐46619, Fluprostenol, sulprostone or Cicaprost (Figure 6c). In Figure 4b, we showed that Dox treatment does not induce 11β‐PGF_2α_, which is a metabolite of PGD_2_. Thus, we conclude that the apical extrusion of YAP (5SA) cells is induced by PGE_2_‐EP2 signaling rather than PGD_2_‐DP signaling. To assess whether PGE_2_ could rescue YAP (5SA) apical extrusion impaired by COX‐2 inhibition, we treated YAP (5SA): MDCK cocultures with PGE_2_ in the presence of NS398. As suggested, PGE_2_ addition restored the apical extrusion of YAP (5SA) cells in a dose‐dependent manner (Figure 6d). These results indicate that PGE_2_ and EP2 are essential for the apical extrusion of YAP (5SA) cells during cell competition.

### 2.7 Adenylyl cyclase‐cAMP‐PKA signaling is essential for the apical extrusion of YAP (5SA) cells

**Figure 7:**
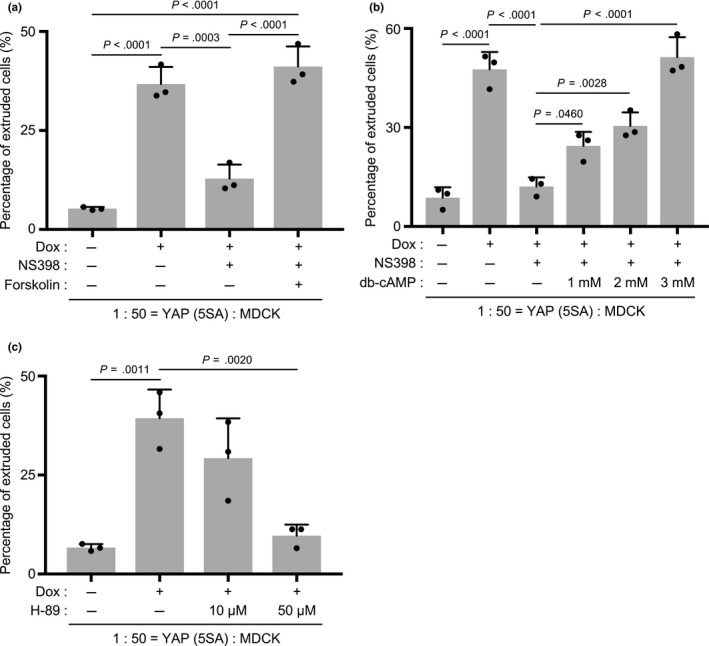
Effects of PKA inhibitor and dibutyryl‐cyclic AMP on the apical extrusion of YAP (5SA) cells. (a–c) Quantification of percentages of apically extruded cells in cocultures of labeled YAP (5SA) cells that were mixed 1:50 with unlabeled normal MDCK cells and treated for 24 hr with Dox plus the indicated compounds starting at 24 hr postseeding. Controls were cocultures incubated for 24 hr with/without Dox in the absence of compounds. (a) Cells were treated with Dox and NS398 (50 μM) plus forskolin (10 μM). (b) Cells were treated with Dox and NS398 (50 μM) plus the indicated concentrations of dibutyryl‐cAMP (db‐cAMP). (c) Cells were treated with Dox plus the indicated concentrations of H‐89 (PKA inhibitor). For (a–c), data are the mean +* SD* (*n* = 3/group) of three independent experiments

EP2 is known as a Gαs‐coupled seven‐spanning receptor that activates adenylyl cyclase (AC), resulting in cyclic AMP (cAMP) production and protein kinase A (PKA) activation (Regan, 2003). To investigate whether this pathway downstream of EP2 was involved in YAP (5SA) apical extrusion, we examined the effects of forskolin, which stimulates AC activity, and dibutyryl‐cAMP (db‐cAMP), which mimics the action of cAMP, on the impairment of YAP (5SA) apical extrusion imposed by COX‐2 inhibition. Both forskolin and db‐cAMP restored the apical extrusion of cocultured YAP (5SA) cells (Figure 7a,b). Next, to determine whether PKA was involved in YAP (5SA) apical extrusion, we treated cocultures with H‐89, a PKA inhibitor, at 24 hr post‐Dox. H‐89 completely blocked the apical extrusion of cocultured YAP (5SA) cells (Figure 7c), confirming that this process depends on AC‐cAMP‐PKA signaling.

### 2.8 EP2 in both YAP (5SA) cells and neighboring normal MDCK cells is essential for YAP (5SA) apical extrusion during cell competition

**Figure 8:**
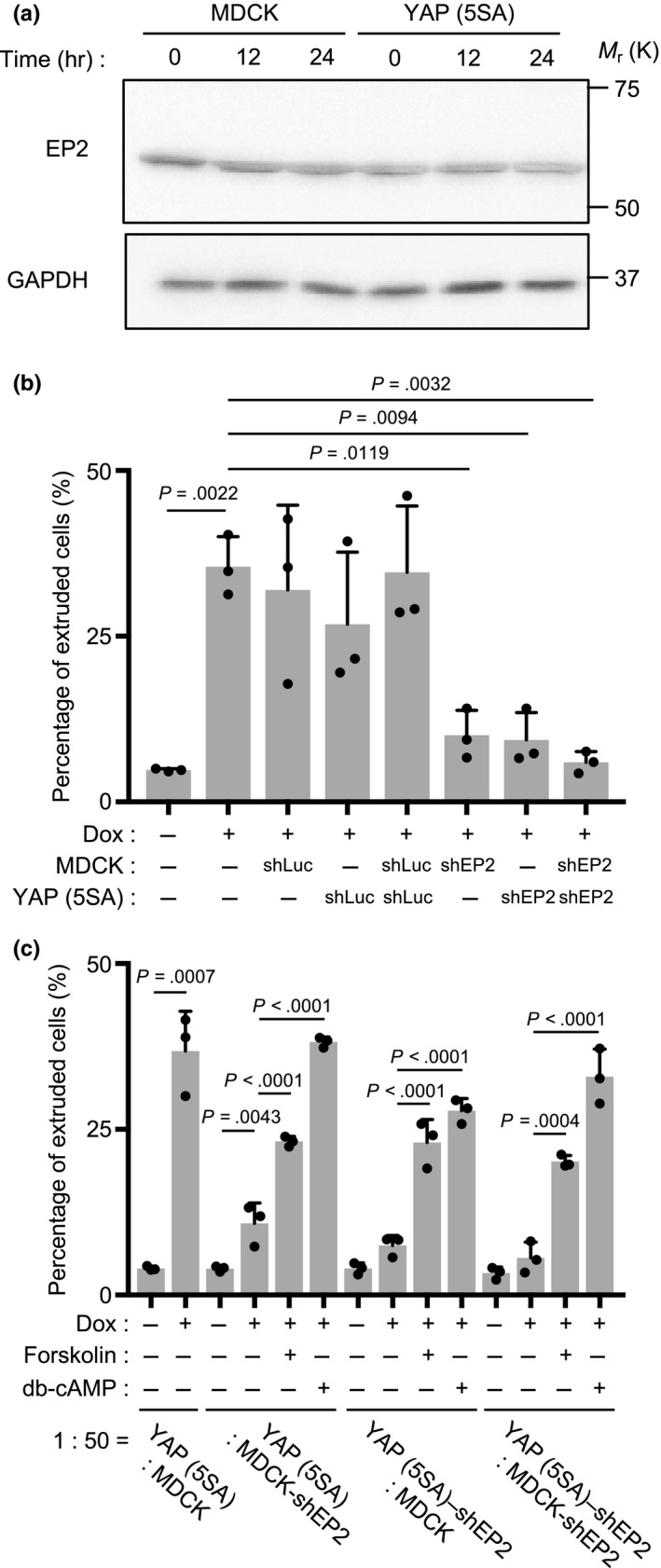
Effects of EP2 knockdown on the apical extrusion of YAP (5SA) cells. (a) Immunoblot to detect EP2 protein in normal MDCK cells and YAP (5SA) cells at 0, 12 and 24 hr after Dox treatment. GAPDH, loading control. (b,c) Quantitation of percentages of apically extruded cells in cocultures of labeled YAP (5SA) cells, or YAP (5SA) cells expressing Luciferase shRNA (shLuc; control), or EP2 shRNA (shEP2), that were mixed 1:50 with unlabeled normal MDCK cells, or MDCK cells expressing shLuc or shEP2, as indicated. Cocultures were treated for 24 hr with Dox starting at 24 hr postseeding. (c) The EP2 knockdown cocultures in (b) were treated for 24 hr with Dox plus forskolin (10 μM) or dibutyryl‐cAMP (3 mM) (as indicated) starting at 24 hr postseeding. For (b) and (c), data are the mean +* SD* (*n* = 3/group) of three independent experiments

Immunoblotting to detect EP2 protein in normal MDCK cells and YAP (5SA) cells showed that EP2 was expressed at equivalent levels in normal MDCK cells and YAP (5SA) cells before Dox treatment and that EP2 was not further increased by YAP activation (Figure 8a). To investigate the importance of EP2 in neighboring MDCK cells for YAP (5SA) cell apical extrusion, we carried out shRNA‐mediated EP2 knockdown experiments in which MDCK and YAP (5SA) cells were transfected with a vector expressing Dox‐inducible EP2 receptor shRNA, and then cocultured in various combinations of transfected and nontransfected cells. We found that EP2 knockdown in surrounding normal MDCK cells inhibited the apical extrusion of nontransfected YAP (5SA) cells, as expected (Figure 8b). To our surprise, however, EP2 knockdown in YAP (5SA) cells also reduced their apical extrusion. To determine whether AC‐cAMP activation could overcome the block imposed by EP2 knockdown, we assessed the effects of forskolin or db‐cAMP treatment on cocultures involving EP2 knockdown cells. Strikingly, the addition of forskolin or db‐cAMP rescued the apical extrusion of YAP (5SA) cells regardless of whether EP2 knockdown had occurred in the normal MDCK cells or YAP (5SA) cells in a coculture (Figure 8c). These results indicate that the apical extrusion of YAP (5SA) cells during cell competition depends on EP2‐AC‐cAMP signaling not only in the YAP (5SA) cells but also in the surrounding normal MDCK cells.

### 2.9 E‐cadherin internalization specifically observed in YAP (5SA) cells is regulated by EP2‐mediated PKA signaling in both neighboring MDCK cells and YAP (5SA) cells

**Figure 9:**
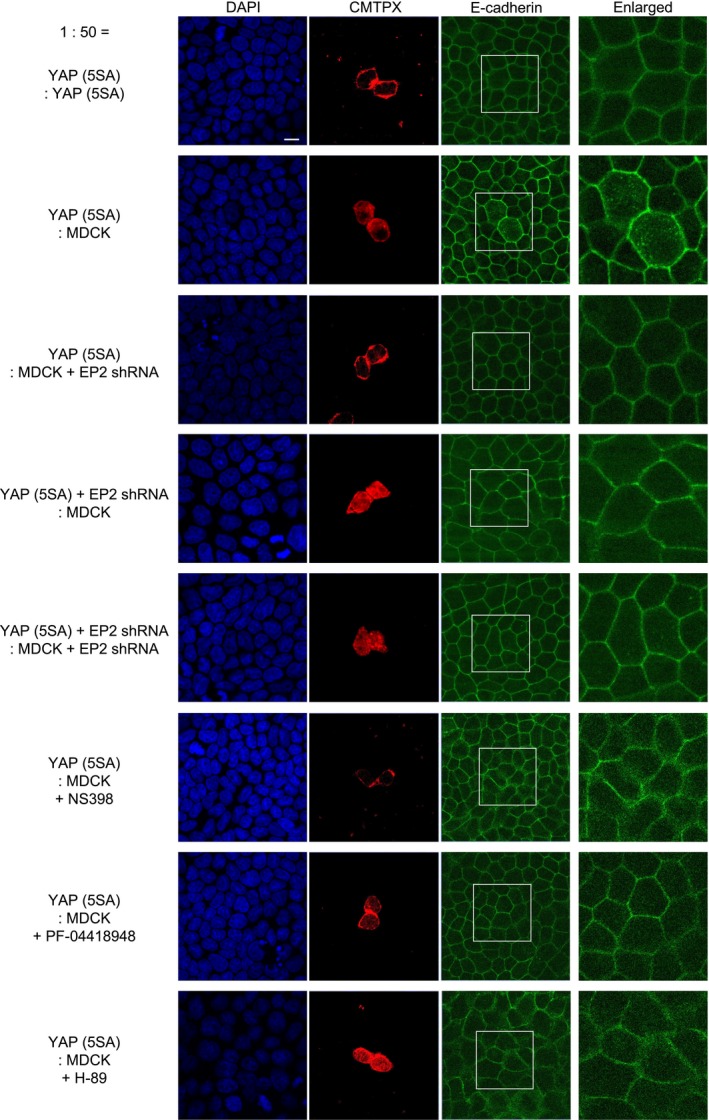
Effects of COX‐2, EP2 and PKA inhibitors on E‐cadherin internalization in YAP (5SA) cells during cell competition. Representative images of immunofluorescent staining of DAPI (blue; nuclei), E‐cadherin (green) and CMTPX (red; labeled YAP (5SA) cells or YAP (5SA) cells expressing shEP2). Labeled YAP (5SA) cells or labeled YAP (5SA) cells expressing shEP2 were mixed 1:50 with unlabeled normal MDCK cells or unlabeled MDCK cells expressing shEP2, as indicated. At 24 hr postseeding, cocultures were treated for 22 hr with Dox plus NS398 (50 μM), PF‐04418948 (10 μM) or H‐89 (50 μM). “Enlarged,” high magnification images of the inset boxes in the E‐cadherin panels. Results are representative of three trials. Scale bar, 10 μM

We previously showed that Ras (G12V)‐expressing MDCK cells undergoing apical extrusion exhibit E‐cadherin internalization (Saitoh et al., 2017). To investigate whether E‐cadherin internalization was also observed during the apical extrusion of YAP (5SA) cells, we carried out E‐cadherin immunostaining and detected E‐cadherin internalization in YAP (5SA) cells surrounded by normal MDCK cells, but not in YAP (5SA) cells surrounded by YAP (5SA) cells (Figure 9). Next, to assess whether EP2 signaling in neighboring normal MDCK cells and/or in YAP (5SA) cells was needed for this E‐cadherin internalization, we carried out EP2 knockdown experiments. Intriguingly, we found that E‐cadherin internalization was inhibited not only when EP2 was depleted in normal MDCK cells, but also when it was depleted in YAP (5SA) cells. To determine whether E‐cadherin internalization was regulated by EP2 signaling, we applied NS398, PF‐04418948 or H‐89 to YAP (5SA): MDCK cocultures and observed that E‐cadherin internalization was inhibited by all three inhibitors. Thus, EP2 expression by neighboring normal MDCK cells is essential for E‐cadherin internalization in YAP (5SA) cells. Furthermore, this E‐cadherin internalization is induced by EP2‐mediated PKA signaling in both neighboring normal MDCK cells and YAP (5SA) cells.

## 3 DISCUSSION

Our study has established that, during competitions between YAP (5SA) (abnormal) cells and surrounding normal MDCK cells, it is PGE_2_‐EP2 signaling that triggers the apical extrusion of the “losing” YAP‐expressing cells. We speculate that COX‐2‐mediated PGE_2_ secretion engages and stimulates EP2 receptors on both neighboring normal cells and the YAP (5SA) cell. The surrounding normal cells are thus alerted to the presence of the abnormal YAP (5SA) cells and initiate cell competition. Within both the normal and YAP (5SA) cells, AC‐cAMP‐PKA signaling downstream of EP2 engagement induces cytoskeleton remodeling. However, only within the YAP (5SA) cells does the activated YAP trigger the E‐cadherin internalization that eventually leads to apical extrusion. Thus, PGE_2_ signaling appears to function as a warning signal of the presence of abnormal cells in a surrounding normal “cell society.”

At the organismal level, PGE_2_ is a lipid mediator that acts to maintain tissue homeostasis. During acute inflammation, PGE_2_ is synthesized and secreted locally to recruit immune cells and promote their infiltration into the affected area (Park et al., 2006; Ricciotti & FitzGerald, 2011; Serhan & Levy, 2003). Secreted PGE_2_ is rapidly metabolized to an inactive form (15‐keto‐prostaglandin) by 15‐hydroxyprostaglandin dehydrogenase (15‐PGDH) (Kochel & Fulton, 2015), limiting the functions of PGE_2_ to an area very near the PGE_2_‐secreting cells. These features make PGE_2_ eminently suitable for mediating the cell competition signaling necessary when normal cells recognize abnormal cells in the immediate vicinity.

Actin polymerization and the formation of intermediate filaments are essential for the apical extrusion of YAP (5SA) cells (Chiba et al., 2016). These processes are both regulated by RhoA phosphorylation and mediated by PKA (Shabb, 2001). We observed that PKA signaling and cytoskeleton remodeling occurred in both surrounding normal MDCK and YAP (5SA) cells during cell competition, but that E‐cadherin internalization took place only in YAP (5SA) cells. Previous reports have shown that the endocytosis of E‐cadherin is induced by the activation of the small GTP‐binding proteins CDC42 and Rac (Akhtar & Hotchin, 2001; Izumi et al., 2004) and that YAP activation induces the expression of various small GTP‐binding protein regulators such as ARHGAP18, ARHGAP29, Ect2 and Fgd3 (Miyamura et al., 2017; Porazinski et al., 2015; Qiao et al., 2017). We thus suggest that, in the presence of PKA‐promoted cytoskeleton remodeling, YAP‐driven regulation of small GTP‐binding proteins may trigger E‐cadherin internalization and thus induce apical extrusion specifically in YAP (5SA) cells.

Recently, studies in *Drosophila* identified Sas‐PTP10D signaling as a mechanism by which normal cells recognize abnormal cells during cell competition and induce them to undergo apoptosis (Yamamoto, Ohsawa, Kunimasa, & Igaki, 2017). Within the normal cells, the ligand Sas is relocalized to the lateral cell surface, whereas within the polarity‐deficient abnormal cells, the receptor‐type tyrosine phosphatase PTP10D is relocalized to the lateral cell surface. The binding of Sas to PTP10D then induces signaling that triggers the apoptosis of the polarity‐deficient cells. Thus, Sas‐PTP10D signaling serves as a sign to normal cells that they should kill the cells that are abnormal due to loss of polarity. In mammals, a PTP10D homologue called PTPRJ exists and contributes to cell contact‐mediated growth inhibition (Qiao et al., 2017; Yamamoto et al., 2017), but, to our knowledge, there has been no report to date on a mammalian Sas homologue (Hendriks & Pulido, 2013; Ostman, Yang, & Tonks, 1994). From these observations, we conclude that several different signaling pathways can be involved in cell competition to maintain tissue homeostasis, with the identity of the pathway depending on the nature of the failing in the abnormal cell. For example, Sas‐PTP10D signaling is used specifically in cell competition to remove polarity‐deficient *Drosophila* cells, but this pathway does not appear to function during any other cell competition in either *Drosophila* or mammals. Similarly, PGE_2_–EP2 signaling is essential for the apical extrusion of mammalian YAP (5SA) cells, but not Ras (G12V) or v‐Src cells. Our study has thus contributed significantly to expanding our knowledge of the cell competition field, and future delving into the elimination mechanisms associated with a range of mammalian cell abnormalities may yield a rich trove of potential therapeutic targets.

## 4 EXPERIMENTAL PROCEDURES

### 4.1 Antibodies and inhibitors

Rabbit anti‐EP2 (No. 101750) polyclonal antibody (Ab) was purchased from Cayman Chemical Company. Mouse anti‐GAPDH (CA92590) Ab was from Merck Millipore. Rat anti‐E‐cadherin (ab11512) Ab was from Abcam. The chemical inhibitors PF‐4708671 (PZ0143, 10 μM), indomethacin (I7378, 10–20 μM), L‐798106 (L4545, 10 μM), forskolin (F3917, 10 μM) and N^6^,2′‐O‐Dibutyryladenosine 3′,5′‐cyclic monophosphate sodium salt (db‐cAMP) (D0627, 1–3 mM) were all purchased from Sigma‐Aldrich; NS398 (ab120295, 10–50 μM) and H‐89 (ab143787, 10–50 μM) were from Abcam; PGE_2_ (163‐10814, 1–5 μM) was from Wako; SC‐560 (70340, 10–50 μM), ONO‐8711 (14070, 10 μM), PF‐04418948 (15016, 10 μM), butaprost (13740, 10 μM), U‐46619 (16450, 100 nM), 15(s)‐Fluprostenol (16787, 10 μM), sulprostone (14765, 10 μM), BW245C (12050, 10 μM) and Cicaprost (16831, 10 μM) were all from Cayman Chemical Company; ONO‐AE3‐208 (CS‐0315, 10 μM) was from Chemscene; and LY294002 (10 μM) and rapamycin (0.1 μM) were from Calbiochem.

### 4.2 Cell lines and culture conditions

Madin‐Darby canine kidney cells expressing YAP (WT), YAP (5SA), K‐Ras (G12V) or v‐Src in a doxycycline (Dox)‐dependent manner were established in a previous study (Chiba et al., 2016). The expression of YAP (WT), YAP (5SA), K‐Ras (G12V) or v‐Src cDNA was induced by the addition of 2 μg/ml doxycycline hydrochloride (Dox; Apollo Scientific Ltd.) to the culture medium.

EP2 knockdown experiments used MDCK cells stably expressing EP2 shRNA or control shRNA in a Dox‐dependent manner. Oligomers used were as follows: Luciferase shRNA oligo‐1, 5′‐CCGGTGAAACGATATGGGCTGAACTCGAGTTCAGCCCATATCGTTTCATTTTT‐3′; Luciferase shRNA oligo‐2, 5′‐AATTAAAAATGAAACGATATGGGCTGAACTCGAGTTCAGCCCATATCGTTTCA‐3′; EP2 shRNA oligo‐1, 5′‐CCGGGACTTCCTGTTCTATACAGTCAAACGCCACTCGAGTGGCGTTTGACTGTATAGAACAGGAAGTCTTTTT‐3′; EP2 shRNA oligo‐2, 5′‐AATTAAAAAGACTTCCTGTTCTATACAGTCAAACGCCACTCGAGTGGCGTTTGACTGTATAGAACAGGAAGTC‐3′. Constructs annealing to the above oligomers were cloned into the Tet‐pLKO‐puro vector (#21915, Addgene). For the generation of lentivirus preparations, subconfluent 293T packaging cells plated on a 10‐cm dish were transfected with either the Tet‐pLKO‐puro‐shLuciferase (shLuc) or Tet‐pLKO‐puro‐shEP2 construct, plus the pRSV‐Rev, pMDLg/pRRE and pMD2.G (pVSV) vectors in the presence of FuGENE HD Transfection Reagent (E231A, Promega). At 24 hr post‐transfection, the culture medium was changed to fresh Dulbecco's modified Eagle's medium [DMEM (Nissui Pharmaceutical Co.)] supplemented with 20% FBS, 2% glutamine, 0.2% sodium hydrogen carbonate, 100 units/ml penicillin and 0.1 mg/ml streptomycin (Sigma). At 24 hr after changing the medium, the supernatants containing lentivirus were collected and used for infection of normal MDCK or YAP (5SA) cells that had been plated at 1 × 10^5^ cells/well in 6‐well plates. Infected cells were cultured for 1 week in the presence of puromycin (final concentration 2 μg/ml) to select for resistance. The expression of shLuc or shEP2 was induced by Dox as described above.

Standard culture and maintenance of all MDCK‐based cell lines occurred in DMEM supplemented with 10% FBS, 2% glutamine, 0.2% sodium hydrogen carbonate, 100 units/ml penicillin and 0.1 mg/ml streptomycin. For passages, cells were washed twice in PBS and treated with 0.05% trypsin solution (trypsin, sodium chloride, potassium chloride, sodium hydrogen phosphate, potassium dihydrogen phosphate, glucose, phenol red) supplemented with 1% EDTA. After incubation for 5 min, cells were resuspended and aliquotted into new culture dishes.

### 4.3 Standard cell competition assay

Type I collagen was obtained from Nitta Gelatin (Nitta Cellmatrix type 1‐A) and neutralized on ice to a final concentration of 2 mg/ml according to the manufacturer's instructions. Triple‐well glass base dishes (3970‐103, IWAKI) or multiwell glass‐bottom dishes (D141400, Matsunami) were coated with 12 μl neutralized collagen and allowed to solidify for 30 min at 37°C. MDCK cells expressing YAP (5SA), K‐Ras (G12V) or v‐Src were mixed with normal MDCK cells at a ratio of 1:50, and the cell mixture was plated at 10–12 × 10^4^ cells/well onto the collagen matrix. After incubation for 24 hr at 37°C, 2 μg/ml Dox was added to induce cDNA expression. After incubation for another 24 hr, the Dox‐treated cells on collagen were fixed with 4% paraformaldehyde/PBS for 10 min at 37°C and washed twice in PBS. Cells were then incubated with Hoechst stain and phalloidin/0.05% Triton X‐100/PBS for 30 min to see apical extrusion.

In experiments where the percentage of extruding cells was to be quantified, MDCK cells expressing YAP (WT), YAP (5SA), K‐Ras (G12V) or v‐Src were labeled with the red fluorescent dye CMTPX **(**Invitrogen) according to the manufacturer's instructions before mixing 1:50 with unlabeled normal MDCK cells. Confocal microscopy was used to count the number of labeled extruding cells and the number of labeled nonextruding cells in the same coculture. The percentage of extruding cells was calculated as the number of labeled extruding cells divided by the total number of labeled cells (extruding plus nonextruding) × 100%. At least 100 labeled cells were counted per culture.

### 4.4 Chemical compound screening and aggregate formation

A library of chemical compounds (FKL series, the Chemical Biology Screening Centre of Tokyo Medical and Dental University) consisting of “The US Drug Collection” (MicroSource Discovery Systems) and “The International Drug Collection” (MicroSource Discovery Systems) was provided by the Institute of Biomaterials and Bioengineering of Tokyo Medical and Dental University. All 1,600 chemical compounds were supplied as 100 μM in DMSO stock solutions in 96‐well plates. Type I collagen was obtained from Nitta Gelatin (Nitta Cellmatrix type 1‐A) and neutralized on ice to a final concentration of 2 mg/ml according to the manufacturer's instructions. Each well of a flat‐bottom 96‐well plate (IWAKI) was coated with 10 μl neutralized collagen and allowed to solidify for 30 min at 37°C. MDCK cells expressing YAP (5SA) were labeled with CMTPX as above and mixed with unlabeled normal MDCK cells at a ratio of 1:50 before the mixture was plated at 7 × 10^4^ cells/well onto the collagen matrix. After incubation for 24 hr at 37°C, 2 μg/ml Dox was added to induce cDNA expression. After incubation for 72 hr, Dox‐treated cells on collagen were washed twice in PBS (‐) and fixed with 4% paraformaldehyde/PBS for 10 min at 37°C. Fixed cells were washed twice in PBS (‐). Cell aggregation was then examined using a phase‐contrast fluorescence microscopy.

### 4.5 Immunoblotting

Immunoblotting was carried out as described previously (Chiba et al., 2016). Blots were incubated overnight at 4°C with anti‐EP2 or anti‐GAPDH Abs. Primary Abs were detected by incubation with anti‐rabbit or anti‐mouse peroxidase‐conjugated secondary Abs from Santa Cruz. Proteins were seen using the SuperSignal West Femto Kit (Pierce) and a ChemiDoc XRS system (Bio‐Rad).

### 4.6 Immunofluorescence

Micro‐cover glasses (Matsunami, 18 × 18 mm, 0.12–0.17 mm) were placed in 35‐mm dishes and coated with 1 ml of a collagen matrix. Cell mixtures (typically normal MDCK cells plus YAP (5SA) cells mixed at a ratio of 1:50) were cultured on these collagen matrices for 24 hr at 37°C until a monolayer was formed. Dox (2 μg/ml) was added for 22 hr to induce cDNA expression. Cells were washed three times in PBS, fixed with 4% paraformaldehyde/PBS for 15 min at room temperature and incubated with 0.5% Triton X‐100/PBS for 15 min at room temperature. Fixed cells were blocked for 1 hr in 1% BSA/PBS, followed by incubation with primary Abs for 16 hr at 4°C, and then with Alexa‐488‐conjugated secondary Abs for 1hr at room temperature. Immunostained cells were incubated with Hoechst dye in 1% BSA/PBS for 15 min and mounted with Mowiol on cover glasses (Matsunami, 24 × 60 mm, 0.12–0.17 mm). Immunofluorescent images were captured and analyzed using a LSM710 Zeiss confocal microscopy.

### 4.7 Quantitative LC‐MS/MS analysis

Quantitative LC‐MS/MS analysis was carried out as described previously (Ohba et al., 2018; Okuno et al., 2018). Briefly, normal MDCK cells were mixed with YAP (5SA) cells at a ratio of 1:50 and the mixture was plated at 5 × 10^6^ cells per 6‐cm dish. After incubation for 24 hr at 37°C in standard medium [Dulbecco's modified Eagle's medium (Nissui Pharmaceutical Co.) supplemented with 10% FBS, 2% glutamine, 0.2% sodium hydrogen carbonate, 100 units/ml penicillin and 0.1 mg/ml streptomycin (Sigma)], the medium was changed to serum‐free medium containing 2 μg/ml Dox, with/without 50 μM of the COX‐2 inhibitor NS398. Serum‐free medium was Dulbecco's modified Eagle's medium lacking L‐Gln, **s**odium pyruvate and phenol red (Nacalai) and supplemented with 2% glutamine, 0.2% sodium hydrogen carbonate and 100 units/ml penicillin and 0.1 mg/ml streptomycin (Sigma). After incubation for 24 hr, the conditioned medium was collected into 15‐ml tubes. Ice‐cold methanol (2 ml) was added to 2 ml of collected medium, and this mixture was diluted with water containing 0.1% formic acid to yield a final methanol concentration of 20%. After centrifugation, deuterium‐labeled internal standards were added and the supernatants loaded on Oasis HLB cartridges (Waters, Milford, MA). The column was sequentially washed with water containing 0.1% formic acid, 15% methanol containing 0.1% formic acid and petroleum ether containing 0.1% formic acid. The samples were eluted with 200 μl methanol containing 0.1% formic acid. For RP‐HPLC‐MS/MS, a Shimadzu LC system consisting of four LC‐20AD pumps, a SIL‐20AC autosampler, a CTO‐20AC column oven, a FCV‐12AH six‐port switching valve and a TSQ Quantum Ultra triple quadrupole mass spectrometer equipped with an ESI ion source (Thermo Fisher Scientific) was used. An aliquot of each sample (50 μl) was injected into the trap column, an OptiGuard Mini C18, at a total flow rate of 500 μl/min. Three minutes after sample injection, the valve was switched to introduce the trapped sample to the analytical column, a Capcell Pak C18 MGS3 (Osaka Soda). Separation of lipids was achieved by a linear gradient using water and acetonitrile containing 0.1% formic acid. The total flow rate was 120 μl/min, the column temperature was set at 46°C, and the LC column eluent was introduced directly into a TSQ Quantum Ultra. All compounds were analyzed in a negative ion polarity mode. Eicosanoids were quantified by multiple reaction monitoring (MRM). The MRM transitions monitored were m/z 369 → 245 for 6‐ketoPGF_1α_, m/z 353 → 193 for PGF_2α_, m/z 353 → 193 for 11β‐PGF_2α_ and m/z 351 → 271 for PGE_2_. For accurate quantification, calibration curves were generated for each target eicosanoid using known reference standards and the same isotope‐labeled internal standard. Automated peak detection, calibration and calculation were carried out using the Xcalibur 2.2 software package.

### 4.8 Statistics

The statistical significance was calculated by GraphPad Prism 7. For comparison between two groups, two‐tailed unpaired *t* test was used. For multigroup comparison, one‐way ANOVA with Tukey's multiple comparison test was used.

## CONFLICT OF INTEREST

The authors declare that no competing financial interests exist.

## AUTHOR CONTRIBUTION

E.I., Y.N. and T.O. designed the experiments and generated most of the data. S.K. and M.Y. carried out experiments. H.K., K.K., S.T., T.Y., Y.S., Y.F. and A.S. conceived and designed this study. This manuscript was written by E.I. and H.N. with assistance from the other authors.
