# Supplementary material for: Prostaglandin E2 and its receptor EP2 trigger signaling that contributes to YAP‐mediated cell competition
Source: Genes Cells. 2020 Feb 7;25(3):197–214. doi: 10.1111/gtc.12750 (PMC7078805; doi:10.1111/gtc.12750)
Supplement: Supplementary file 1 [file GTC-25-197-s001.docx]

Supplementary Table 1: List of 55 chemical compounds used in high-throughput screening.

| Number | Compound Name | CAS No. | Clinical & Biological Uses |
| --- | --- | --- | --- |
| 1 | HARMINE | 442-51-3 | Biological use: Antiparkinsonian agent; Active against gram-positive bacteria and fungi, Mechanism: CNS stimulant; MAO-A inhibitor |
| 2 | CHRYSIN | 480-40-0 | Biological use: Shows antifungal activity, Mechanism: Lipid peroxidation inhibitor in rat liver microsomes |
| 3 | MYCOPHENOLATE MOFETIL | 115007-34-6 | immune suppressant, antineoplastic, antiviral, Material source: semisynthtic; RS-61443 |
| 4 | TRIMEBUTINE MALEATE | 34140-59-5 (base) | antispasmodic, opiod receptor agonist, Material source: synthetic |
| 5 | DIBUTYL PHTHALATE | 84-74-2 | plasticiser, suspect endocrine disruptor, Material source: synthetic |
| 6 | BENZALKONIUM CHLORIDE | 8001-54-5 | antiinfective (topical), Material source: synthetic |
| 7 | MYCOPHENOLIC ACID | 24280-93-1 | immune suppressant, antineoplastic, antiviral, Material source: Penicillium brevicompactum and other Penicillium spp |
| 8 | GLUTATHIONE | 70-18-8 | antioxidant, Material source: plant and animal tissue |
| 9 | BUPROPION HYDROCHLORIDE | 31677-93-7 | Biological use: Antidepressant; Used as a smoking cessation aid, Mechanism: Dopamine reuptake inhibitor |
| 10 | LEVONORGESTREL | 797-63-7 | Biological use: Tocolytic; Used in oral contraceptives, Mechanism: Progestogen |
| 11 | TOLBUTAMIDE | 64-77-7 | antidiabetic, Material source: synthetic |
| 12 | PERGOLIDE MESYLATE | 66104-23-2,  66104-22-1 [pergolide] | dopamine receptor agonist, Material source: semisynthetic |
| 13 | FENSPIRIDE HYDROCHLORIDE | 5053-08-7,  5053-06-5 [fenspiride] | antiinflammatory, bronchodilator, Material source: synthetic |
| 14 | RETINYL ACETATE | 127-47-9 | vitamin precursor, Material source: semisynthetic |
| 15 | OXIGLUTATIONE DISODIUM SALT | 103239-24-3; 27025-41-8 (acid) | antioxidant, Material source: synthetic |
| 16 | PHTHALYLSULFATHIAZOLE | 85-73-4 | antibacterial, Material source: synthetic |
| 17 | TRIFLUPROMAZINE HYDROCHLORIDE | 1098-60-8,  146-54-3 [triflupromazine] | antipsychotic, Material source: synthetic |
| 18 | TRIACETIN | 102-76-1 | antifungal (topical), Material source: synthetic |
| 19 | MEFLOQUINE | 53230-10-7 | antimalarial, Material source: synthetic |
| 20 | THIOPENTAL SODIUM | 71-73-8, 76-75-5 [thiopental] | anesthetic, Material source: synthetic |
| 21 | BUPIVACAINE | 38396-39-3 | Biological use: Local anaesthetic, Mechanism: Blocks the generation and the conduction of nerve impulses, presumably by increasing the threshold for electrical excitation in the nerve, by slowing the propagation of the nerve impulse. |
| 22 | ANCITABINE HYDROCHLORIDE | 10212-25-6 | antineoplastic, Material source: synthetic |
| 23 | TOLPERISONE HYDROCHLORIDE | 70312-00-4 | muscle relaxant (skeletal), Material source: synthetic; N-553 |
| 24 | SULFASALAZINE | 599-79-1 | anticolitis and Crohn's disease, Material source: synthetic |
| 25 | GLICLAZIDE | 21187-98-4 | antidiabetic, adhesion inhibitor, Material source: synthetic; SE-1702 |
| 26 | MEDRYSONE | 2668-66-8 | glucocorticoid, Material source: semisynthetic |
| 27 | PROTIONAMIDE | 14222-60-7 | antibacterial, Material source: synthetic; TH-1321, RP-9778 |
| 28 | AMPICILLIN SODIUM | 69-52-3, 69-53-4 [ampicillin] | antibacterial, Material source: semisynthetic |
| 29 | BEZAFIBRATE | 41859-67-0 | antihyperlipidemic, Material source: synthetic |
| 30 | CYTARABINE | 147-94-4 | antineoplastic, antiviral, antimetabolite, Material source: synthetic |
| 31 | BENZOYLPAS | 13898-58-3 | antibacterial, tuberculostatic, Material source: synthetic |
| 32 | PENICILLAMINE | 52-67-5 | chelating agent (Cu), antirheumatic, Material source: semisynthetic |
| 33 | AMENDOL | 5546-17-8 | Biological use: Antidepressant; Psychostimulant,  Mechanism: Serotonin release regulator |
| 34 | PROPANTHELINE BROMIDE | 50-34-0,  298-50-0 [propantheline] | anticholinergic, Material source: synthetic |
| 35 | SULFAMETHIZOLE | 144-82-1 | antibacterial, Material source: synthetic |
| 36 | CARMOFUR | 61422-45-5 | antineoplastic, Material source: synthetic |
| 37 | METHIMAZOLE | 60-56-0 | thyroid inhibitor, Material source: synthetic |
| 38 | CHLORAMPHENICOL | 56-75-7 | Biological use: Antibiotic, Mechanism: Protein synthesis inhibitor; Interferes with transfer of activated aminoacids from soluble RNA to ribosomes |
| 39 | 8-OXOADENINE | 21149-26-8 | Biological use: DNA mismatch in mutagenesis, Mechanism: Induces misincorporation in in vitro DNA synthesis and mutation in NIH 3T3, Inhibits or blocks WRNp |
| 40 | SULFADIAZINE | 68-35-9 | antibacterial, Material source: synthetic |
| 41 | INOSITOL | 87-89-8 | growth factor, Material source: lipotropic polyol widely distributed in plants and animals |
| 42 | SQUAMOLONE | 40451-67-0 | Biological use: Cytostatic, Mechanism: Potent inhibition of mitochondrial NADH |
| 43 | HOMATROPINE BROMIDE | 51-56-9,  87-00-3 [homatropine] | anticholinergic (opthalmic), Material source: semisynthetic |
| 44 | APOMORPHINE HYDROCHLORIDE | 41372-20-7,  314-19-2 [anhydrous],  58-00-4 [apomorphine] | emetic, Material source: synthetic |
| 45 | SALICYLAMIDE | 65-45-2 | analgesic, Material source: synthetic |
| 46 | DYCLONINE HYDROCHLORIDE | 536-43-6,  586-60-7 [dyclonine] | anesthetic (topical), Material source: synthetic |
| 47 | THIABENDAZOLE | 148-79-8 | anthelmintic, Material source: synthetic |
| 48 | ENILCONAZOLE | 35554-44-0 | antifungal, Material source: synthetic; R-23979 |
| 49 | MEFENAMIC ACID | 61-68-7 | antiinflammatory, analgesic, Material source: synthetic |
| 50 | COLISTIMETHATE SODIUM | 8068-28-8,  21362-08-3 [replaced] | antibacterial, Material source: Bacillus colistinus |
| 51 | IOPANIC ACID | 96-83-3 | radioopaque agent, Material source: synthetic |
| 52 | BACITRACIN | 1405-87-4 | antibacterial, Material source: Bacillus licheniformis and B subtilis |
| 53 | HYDROCORTISONE ACETATE | 50-03-3 | glucocorticoid, antiinflammatory, Material source: semisynthetic |
| 54 | GLUCOSAMINE HYDROCHLORIDE | 3416-24-8 | antiarthritic, Material source: polysaccharides in bacteria, fungi, higher plants, invertebrates, vertebrates |
| 55 | GALLAMINE TRIETHIODIDE | 65-29-2, 153-76-4 [gallamine] | muscle relaxant (skeletal), Material source: synthetic |
